# Enhancing Lactate Assimilation in *Aspergillus oryzae* by Small Amounts of Metabolic Co-Substrates and Low pH

**DOI:** 10.3390/jof12090679

**Published:** 2026-09-10

**Authors:** Hiroki Kikuta, Masamichi Muto, Naoko Tanaka, Tsuyoshi Tanaka, Junichi Mano

**Affiliations:** 1 Institute of Food Research, National Agriculture and Food Research Organization, 2-1-12 Kannondai, Tsukuba 305-8642, Ibaraki, Japan; kikuta.hiroki359@naro.go.jp; 2 Microbial Technology Research Section, Biotics Research Institute, R&D Division, Morinaga Milk Industry Co., Ltd., 5-1-83 Higashihara, Zama 252-8583, Kanagawa, Japan; 3 Research Center for Advanced Analysis, National Agriculture and Food Research Organization, 2-1-2 Kannondai, Tsukuba 305-8518, Ibaraki, Japan; 4 Institute of Crop Science, National Agriculture and Food Research Organization, 2-1-2 Kannondai, Tsukuba 305-8518, Ibaraki, Japan; 5 Faculty of Life and Environmental Sciences, University of Tsukuba, 1-1-1 Tennodai, Tsukuba 305-8572, Ibaraki, Japan

**Keywords:** *Aspergillus*, lactic acid, condition-specific metabolism, upcycling, food waste valorization, mycoprotein

## Abstract

Lactate-rich byproducts and fermentation residues are generated during various food and fermentation processes, presenting opportunities for their conversion into value-added products through microbial bioprocesses. *Aspergillus oryzae* is a promising microbial host for such applications because of its ability to use diverse substrates and produce valuable products. However, the ability of *A. oryzae* to assimilate lactate and the factors limiting this process remain poorly understood despite its industrial importance. In this study, we investigated the transcriptional responses of *A. oryzae* during lactate assimilation and evaluated the culture conditions promoting this process. *A. oryzae* demonstrated only limited growth and lactate consumption when lactate was provided as its only carbon source in the synthetic minimal medium. Transcriptome analysis suggested possible metabolic constraints associated with carbon flux into the tricarboxylic acid (TCA) cycle, limiting lactate assimilation. To alleviate these constraints, the culture conditions were examined. Supplementation with small amounts of sugars, specific amino acids, and α-ketoglutarate promoted lactate assimilation. Moreover, low-pH conditions further improved lactate assimilation, reducing residual lactate concentrations to below the detection limit. These findings indicate that efficient lactate assimilation is promoted by small amounts of metabolic co-substrates, potentially through effects on central carbon metabolism, together with favorable low-pH conditions.

## 1. Introduction

The food and fermentation industries generate large amounts of food byproducts and fermentation residues. These materials contain substantial amounts of carbon sources and nutrients and can be used as feedstocks for microbial cultivation. The use of microbial fermentation provides a means of both reducing waste and producing valuable products. In recent years, the development of upcycling technologies that convert industrial byproducts into value-added products has gained increasing attention [1,2].

*Aspergillus oryzae* is a safe microorganism that has long been used in producing traditional Japanese fermented foods [3,4]. The fungus secretes various extracellular enzymes and can utilize diverse food-derived components, including starch, proteins, and polysaccharides [5,6]. The genome sequence of *A. oryzae* has been identified, and information on gene annotation and metabolic pathways is available through databases, including the Kyoto Encyclopedia of Genes and Genomes (KEGG) [7]. Further, genetic engineering techniques, including transformation and gene expression control systems, have been developed in this species [8]. Hence, *A. oryzae* has been used not only for food applications but also as a chassis microorganism in metabolic engineering and synthetic biology studies. Therefore, the fungus has attracted attention as a host for producing industrial enzymes, valuable chemicals, and functional compounds [9]. In recent years, the direct use of fungal biomass as an alternative protein source (mycoprotein) has been explored. Several studies have shown the potential of *A. oryzae* for producing protein-rich fungal biomass from food byproducts [10,11,12,13,14].

**Figure 1:**
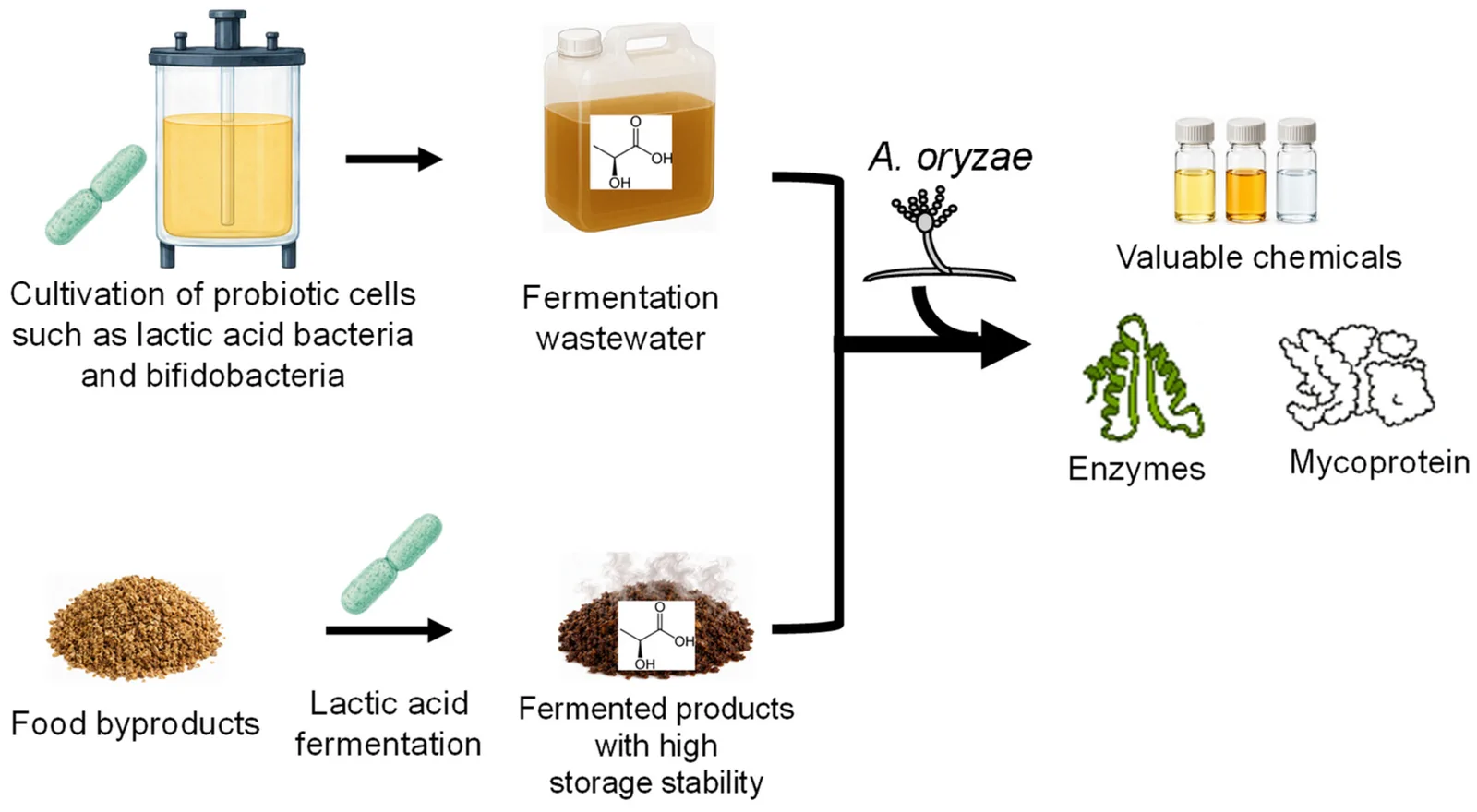
Proposed strategy for upcycling lactate-rich byproducts and fermentation residues using *A. oryzae*. Conceptual illustration of an upcycling approach in which lactate-rich food and fermentation byproducts, including cultivation residues from probiotic microorganisms, are used as substrates for *A. oryzae* cultivation and converted into value-added products. Further, the concept includes the use of food byproducts preserved through lactic acid fermentation to improve their stability.

Among the byproducts generated by the food and fermentation industries, some contain substantial amounts of lactate. Examples include spent media from the cultivation of probiotic microorganisms such as lactic acid bacteria and bifidobacteria, byproducts from fermented dairy production, residues from fermented food manufacturing, and fermentation effluents generated from different fermentation processes [15,16]. Further, food byproducts are frequently susceptible to spoilage during storage, and preservation by acidification through lactic acid fermentation may be an effective approach for improving storage stability [17]. Therefore, it is important to consider the presence of lactate when designing bioprocesses that use food byproducts and fermentation residues as substrates (Figure 1).

These byproducts contain lactate as a potentially available carbon source. However, unlike fermentable sugars such as glucose, lactate can be used by only a limited range of microorganisms, and its assimilation efficiency is strongly influenced by cultivation and nutritional conditions [18,19]. If lactate remains unused, it may increase wastewater treatment costs and environmental burdens. Therefore, the development of microbial conversion technologies that can efficiently use lactate as a carbon source has gained considerable interest. Lactate assimilation has been investigated in various bacteria and yeasts [18,19,20,21,22,23]. In general, lactate is transported into the cell and converted to pyruvate by lactate dehydrogenase, after which it enters central carbon metabolism [22,23]. However, numerous aspects of the physiological conditions and regulatory mechanisms that enable efficient lactate assimilation remain unclear.

Conversely, studies on *A. oryzae* have mainly focused on cultivation and fermentation using carbohydrate substrates, including glucose and starch, and information on lactate use remains limited [12,13,14]. The presence of an L-lactate dehydrogenase (L-LDH) involved in lactate metabolism has been reported in *A. oryzae* [24]. Further, the lactate transporters JenA and JenB have been identified in *Aspergillus nidulans* [25], and homologous genes are predicted to be present in the *A. oryzae* genome. These findings suggest that *A. oryzae* possesses the genetic components required for lactate assimilation. However, previous studies have shown that lactate assimilation by *A. oryzae* is limited [26,27]. Although yeast extract (YE) supplementation has been reported to promote lactate assimilation, only 37.5% of the initial lactate was consumed after 96 h of cultivation, indicating that lactate assimilation remains relatively inefficient [27]. Moreover, because YE is a complex nutrient source containing various components, the nutrients promoting lactate assimilation remain unclear. Furthermore, the metabolic responses associated with lactate use have not been sufficiently characterized. Therefore, elucidating the cultivation conditions and metabolic mechanisms required for efficient lactate assimilation in *A. oryzae* will be important for developing cultivation processes using lactate-containing byproducts as feedstocks. 

In this study, we assessed the lactate assimilation ability of *A. oryzae* and analyzed its metabolic response during lactate utilization by transcriptome analysis. Furthermore, based on the observed transcriptional responses, we inferred potential metabolic constraints on lactate assimilation. Further, we examined nutritional and environmental factors affecting lactate utilization to identify cultivation conditions that promote lactate assimilation.

## 2. Materials and Methods

### 2.1. Strain and Spore Preparation

In this study, the RIB40 strain (NBRC 100959), a representative wild-type strain of koji mold *A. oryzae*, was obtained from the Biological Resource Center of the National Institute of Technology and Evaluation (NBRC, Kisarazu, Japan) and used as the primary strain. Further, multiple *A. oryzae* strains, including RIB128, RIB143, RIB163, RIB301, RIB306, RIB315, RIB915, RIB1108, RIB1172, RIB1178, and OIS1, provided by the National Research Institute of Brewing (Higashihiroshima, Japan), were used where indicated. A spore suspension was prepared as described by Kikuta et al. (2025) [28]. In brief, the RIB40 strain was cultured in Czapek–Dox (CD) medium for 4 days, and the spores were collected using sterile cotton swabs and suspended in an aqueous solution containing 1 g/L Tween 80 and 4 g/L sodium chloride. The suspension was appropriately diluted, and a CO7500 Colorwave spectrophotometer (Biochrom Ltd., Cambridge, UK) was used to measure the optical density at 600 nm (OD_600_). The OD_600_ of the prepared spore suspension was approximately 20–25.

### 2.2. Culture Medium and Basic Culture Conditions

A modified CD medium containing D-glucose or L-lactate as the sole carbon source was used to evaluate the lactate assimilation capacity of *A. oryzae*. The composition of the modified CD medium was as follows: carbon source, 5.0 g/L; ammonium sulfate, 4.7 g/L; potassium chloride, 0.52 g/L; potassium dihydrogen phosphate, 1.52 g/L; magnesium sulfate (MgSO_4_·7H_2_O), 0.246 g/L; ferrous sulfate (FeSO_4_·7H_2_O), 0.0025 g/L; disodium EDTA (EDTA·2Na), 0.025 g/L; zinc sulfate (ZnSO_4_·7H_2_O), 0.011 g/L; boric acid (H_3_BO_3_), 0.0055 g/L; manganese chloride (MnCl_2_·4H_2_O), 0.0025 g/L; cobalt chloride (CoCl_2_·6H_2_O), 0.0008 g/L; copper sulfate (CuSO_4_·5H_2_O), 0.0008 g/L; and ammonium molybdate [(NH_4_)_6_Mo_7_O_24_·4H_2_O], 0.00055 g/L. The glucose and lactate media were referred to as Glu and Lac, respectively. The Lac medium was utilized as the basal medium. Sugars, sugar alcohols, amino acids, α-ketoglutarate (α-KG), or YE were added to the Lac medium at final concentrations of 0.2–1.0 g/L to investigate the culture conditions that promote lactate assimilation. Unless otherwise specified, the pH was adjusted before sterilization, and the media were sterilized via autoclaving, leading to a final pH of 5.8.

For cultivation, 2 mL of medium was dispensed into 14-mL polypropylene round-bottom tubes with two-position caps (Corning Inc., Corning, NY, USA). Each tube was inoculated with 20 μL of spore suspension and incubated with the caps loosely closed at 30 °C and 200 rpm for 24–120 h. Cultivations were generally performed in triplicate.

### 2.3. Evaluation of Glucose and Lactate Consumption

Five hundred microliters of the culture medium before inoculation and the supernatant after cultivation were collected into a 1.5 mL tube. Amberlite IR-120B (Organo Corp., Tokyo, Japan) was added as a cation-exchange resin. The samples were centrifuged at approximately 21,000× *g* for 40 s after vortexing. A 0.22 μm hydrophilic nylon syringe filter (Hawach Scientific Co., Ltd., Xi’an, China) was used to filter the obtained supernatant, and high-performance liquid chromatography (HPLC) was used to analyze the prepared samples. A Shimadzu Corporation LC-VP system was used for HPLC analysis, featuring a differential refractive index detector model RID-10A from the same manufacturer (Shimadzu Corp., Kyoto, Japan). The mobile phase consisted of 14 mM H_2_SO_4_ and was delivered through an Aminex HPX-87H column (300 × 7.8 mm; Bio-Rad, Hercules, CA, USA) at a flow rate of 0.7 mL/min. The column oven and refractive index detector were maintained at 60 °C and 40 °C, respectively. A 10 μL aliquot of each sample was injected using an SIL-10ADvp autosampler (Shimadzu Corp.). LabSolutions version 5.73 (Shimadzu Corp.) was used for data analysis. The HPLC peak area obtained from a medium without carbon sources was used as the background value. For each culture condition, the medium was analyzed prior to inoculation, and the peak area corresponding to the initial glucose or lactate concentration in the prepared medium was determined. Residual glucose and lactate concentrations in the culture medium were calculated using the resulting peak area. Statistical analyses were performed using one-way analysis of variance (ANOVA) followed by Tukey’s honest significant difference (HSD) test, where appropriate.

### 2.4. Estimation of Dry Cell Weight

It was difficult to evaluate the dry cell weight in small-scale experiments; thus, the nucleic acid content of the culture broth was evaluated as an indicator of fungal growth [29]. After collecting the samples for HPLC, 60% (*w*/*w*) perchloric acid was added to the remaining culture containing the cells at one-twentieth of the culture volume. The mixture was heated at 95 °C for 15 min to extract nucleic acids. The mixture was briefly vortexed, and the absorbance at 260 nm (A_260_) of the supernatant after centrifugation was measured using a NanoDrop spectrophotometer (Thermo Fisher Scientific, Waltham, MA, USA) to quantify the nucleic acid content (A_260_s). The culture medium before inoculation was processed similarly and used as a background control (A_260_b).

A pre-prepared calibration curve was used to estimate the dry cell weight from the nucleic acid content. Suspensions containing 0, 0.8, 1.2, 1.8, 2.7, 4.0, 4.9, and 7.2 g/L freeze-dried *A. oryzae* biomass cultivated in CD medium at 30 °C and 200 rpm for 2 days under the same 2 mL culture conditions described above were used to generate the calibration curve. These suspensions were subjected to the same perchloric acid extraction procedure, followed by A_260_ measurement (Figure S1). According to the resulting calibration curve, the estimated dry cell weight (EDCW, g/L) was calculated using the following equation:

EDCW = [(A_260_s) − (A_260_b)]/1.6216, (1)

However, this method cannot be employed in media containing YE, presumably because the background absorbance at 260 nm (A_260_b) is excessively high due to the presence of nucleic acids derived from YE. Instead, after cultivation, the culture supernatant was removed, and the mycelia were washed twice with distilled water. Subsequently, 2 mL of distilled water was added, and the samples were subjected to the same perchloric acid extraction procedure to estimate EDCW. In contrast, the contribution of background absorbance was relatively small in the other media conditions and was corrected by subtracting the A_260_ value of the uninoculated medium (A_260_b).

Although EDCW provides a practical indicator for comparing fungal growth among various culture conditions, it may be influenced by variations in nucleic acid content associated with culture conditions and growth stage. Therefore, EDCW should be regarded as an estimated measure of biomass rather than an absolute measure of biomass. Statistical analyses were performed using one-way analysis of variance (ANOVA) followed by Tukey’s honest significant difference (HSD) test, where appropriate.

### 2.5. RNA-Seq and Downstream Data Analysis

*A. oryzae* RIB40 was cultured under three conditions for RNA-seq analysis: Glu medium for 48 h, Lac medium for 96 h, and Lac medium supplemented with 1 g/L glucose for 72 h (Lac + 1 g/L Glu). Cultivation was performed in 500-mL Erlenmeyer flasks containing 100 mL of medium at 30 °C with shaking at 100 rpm. Sampling time points were selected to compare cultures in which different carbon sources were being actively utilized and fungal growth was at a comparable level. Under the Glu condition, samples were collected during active glucose utilization. Under the Lac + 1 g/L Glu condition, samples were collected during lactate utilization following glucose depletion. In contrast, under the Lac condition, mycelia were harvested after 96 h because sufficient biomass for RNA-seq analysis could not be obtained at earlier time points. Mycelia were harvested after cultivation and used for RNA extraction. RNA extraction, RNA quality assessment, library preparation, and sequencing were performed as described by Kikuta et al. (2025) [28]. Three independent biological replicates were analyzed under each condition (*n* = 3). The RNA-seq data generated in this study were deposited in the DDBJ Sequence Read Archive (DRA) under BioProject accession number PRJDB42398.

The nf-core/RNA-seq pipeline (version 3.13.0) with the default parameters was used for RNA-seq data analysis [30]. The pipeline performed quality control, adapter trimming, read filtering, alignment to the reference genome, and gene expression quantification. The reference genome and annotation of *A. oryzae* RIB40 (GCF_000184455.2) were used in this study. Table S1 provides the gene count matrices generated by the pipeline and used for downstream analyses.

Differential expression analysis was conducted using DESeq2 (version 1.50.2) in R (version 4.5.2) [31]. Samples were grouped based on culture conditions (Glu, Lac, and Lac + 1 g/L Glu). Before analysis, genes with total read counts of <10 across all samples were excluded. Differential expression analysis was conducted using the standard DESeq2 workflow, which includes library size normalization, dispersion estimation, and statistical testing. For each gene, log2 fold change (log2FC) values and adjusted *p*-values (*p*adj; Benjamini–Hochberg method) were obtained. Differentially expressed genes (DEGs) were defined as those with *p*adj of <0.05 and |log2FC| of >1. Differential expression analyses were separately conducted for Glu vs. Lac and Lac vs. Lac + 1 g/L Glu.

KEGG pathway enrichment analysis was conducted using the DEGs identified in the Glu vs. Lac comparison. DEGs were categorized into upregulated genes (log2FC > 1), downregulated genes (log2FC < −1), and all DEGs (|log2FC| > 1), and enrichment analyses were conducted for each group. *A. oryzae* gene (AO) ID and KEGG ortholog (KO) terms were mapped using the KEGG REST API (https://rest.kegg.jp/link/ko/aor; accessed on 29 May 2026) (Table S2). A total of 4465 KO term assignments were identified in *A. oryzae*. For enrichment analysis, 2034 unique KO terms were used as the background set. Enrichment analysis was conducted in R (version 4.5.3) using the clusterProfiler package (version 4.18.4) with the enrichKEGG function [32]. KEGG pathways with a false discovery rate (FDR)-adjusted *p*-value of <0.05 were significantly enriched. Enrichment results were visualized using the R package enrichplot (version 1.30.4) [33]. Dot plots were generated according to the GeneRatio, Count, and *p*-value.

KO terms from enriched KEGG pathways were extracted and mapped onto metabolic pathways according to functional information. AO IDs corresponding to these KO terms were determined, and their gene counts were subjected to variance-stabilizing transformation (VST) using DESeq2 to stabilize the variance across the range of gene expression levels, thereby decreasing the dependence of variance on expression level and facilitating comparison and visualization of gene expression profiles under Glu, Lac, and Lac + 1 g/L Glu conditions. Statistical significance between Glu and Lac conditions and between Lac and Lac + 1 g/L Glu conditions was identified using *p*adj obtained from the corresponding differential expression analyses.

## 3. Results

### 3.1. Comparison of Glucose and Lactate Metabolism in Aspergillus oryzae

**Figure 2:**
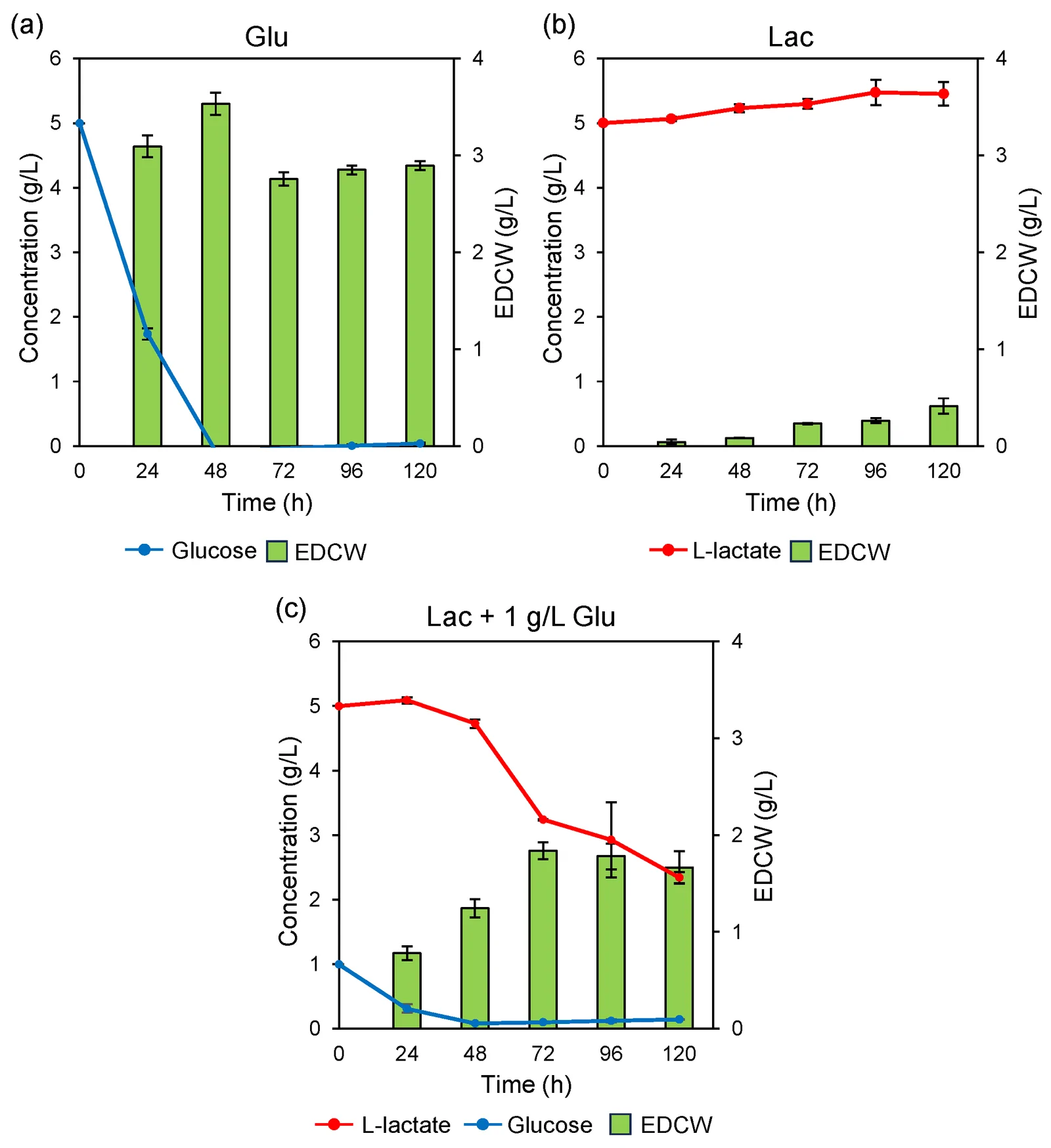
Comparison of glucose and lactate assimilation abilities of *A. oryzae*. Time-course profiles of fungal cell growth and carbon source consumption by *A. oryzae* RIB40 under Glu (**a**), Lac (**b**), and Lac + 1 g/L Glu (**c**) conditions. Green bars indicate EDCW, and lines indicate residual carbon source concentrations. Blue and red lines represent residual glucose and lactate concentrations, respectively. Data are presented as mean ± standard deviation (SD) of three biological replicates (*n* = 3).

*A. oryzae* RIB40 is a representative reference strain whose genome sequence and gene annotations have been extensively characterized [7]. To assess the ability of *A. oryzae* to assimilate lactate as the sole carbon source, the strain was cultivated in a medium containing 5 g/L glucose (Glu) or 5 g/L lactate (Lac). Under the Glu condition, glucose was completely consumed within 48 h of cultivation, leading to rapid fungal growth and the formation of mycelial pellets, with an EDCW reaching a maximum of 3.5 ± 0.1 g/L (Figure 2a). Conversely, under the Lac condition, lactate was not consumed throughout the cultivation period, and only a limited increase in EDCW was observed (Figure 2b). Dense pellet formation was not observed even after 120 h of cultivation, and only very small mycelial aggregates were present. These results indicate that *A. oryzae* cannot efficiently assimilate lactate as the sole carbon source.

**Figure 3:**
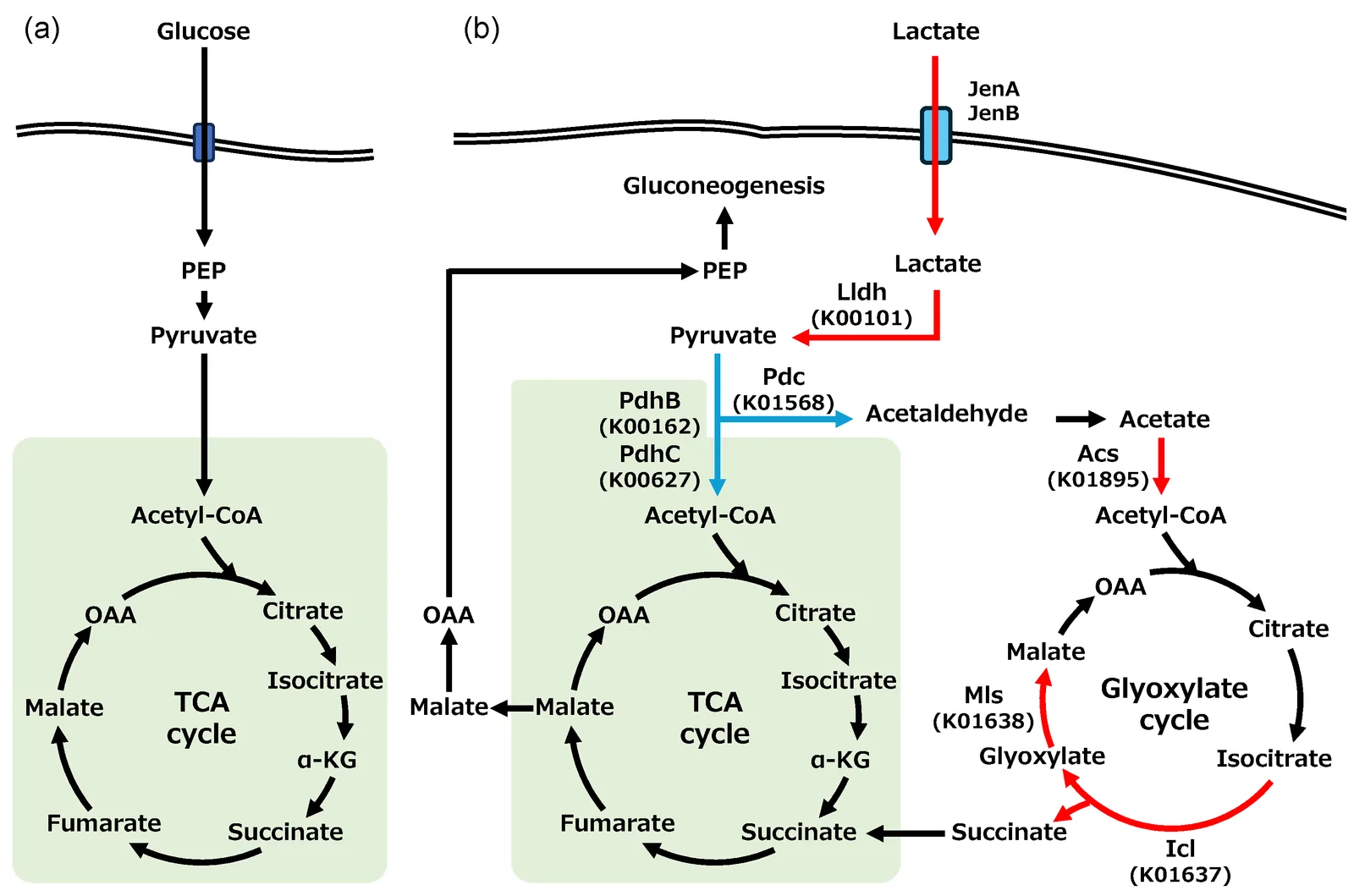
Mapping transcriptional responses to lactate in *A. oryzae*. (**a**) Schematic representation of central carbon metabolism under the Glu condition. (**b**) Transcriptional responses under the Lac condition mapped onto the corresponding metabolic pathways based on KEGG enrichment analysis. The red and blue arrows indicate enzymatic functions assigned to KO terms classified as upregulated and downregulated under the Lac condition, respectively. The classification of KO terms was based on the KEGG enrichment analysis shown in Table S6. The pathway map was adapted and modified from [22,34].

Under the Glu condition, glucose is generally metabolized via glycolysis to pyruvate and subsequently enters the tricarboxylic acid (TCA) cycle (Figure 3a). Conversely, lactate is classified as a non-fermentable carbon source, together with glycerol, ethanol, and acetate. Studies in *Saccharomyces cerevisiae* have shown that assimilation of these substrates is accompanied by extensive transcriptional reprogramming, including the induction of genes involved in gluconeogenesis, the glyoxylate cycle, and the TCA cycle [22,23]. Following cellular uptake, lactate is converted to pyruvate and subsequently used through these central metabolic pathways for energy generation and fungal cell growth. According to this established metabolic framework, Figure 3a,b illustrate the putative carbon metabolic pathways operating during glucose and lactate utilization, respectively.

To characterize transcriptional responses associated with lactate assimilation in *A. oryzae*, RNA-seq analysis was conducted using mycelia cultured under Glu and Lac conditions. Differential expression analysis was conducted on 12,347 genes annotated in *A. oryzae* RIB40, and log2FC and *p*adj were calculated for all genes (Table S3). Based on a threshold of *p*adj of <0.05 and |log2FC| of >1, 3415 genes were identified as DEGs between the Glu and Lac conditions (Table S4). These results indicate that the Lac condition induces a markedly different transcriptional response compared with the Glu condition. To comprehensively characterize the biological functions associated with the observed transcriptional response, KEGG pathway enrichment analysis was conducted using all identified DEGs (Figure S2 and Table S5). As a result, broad metabolic categories, such as “biosynthesis of secondary metabolites,” “microbial metabolism in diverse environments,” “biosynthesis of amino acids,” “carbon metabolism,” and “biosynthesis of cofactors,” were highly represented. In addition to these broad functional categories, genes classified into more specific metabolic pathways, including “glycolysis/gluconeogenesis,” “pyruvate metabolism,” and “glyoxylate and dicarboxylate metabolism,” were also significantly enriched. This observation is consistent with previous studies in *S. cerevisiae*, in which assimilation of non-fermentable carbon sources, including lactate, is accompanied by transcriptional reprogramming of central carbon metabolism [22,23].

To further investigate the transcriptional responses within the pathways associated with lactate assimilation, KO terms contributing to their enrichment were extracted, and their functional roles were examined. KO terms associated with lactate assimilation and carbon flux toward the TCA cycle, including L-lactate dehydrogenase (Lldh; K00101), pyruvate dehydrogenase E1 component subunit beta (PdhB; K00162), and pyruvate dehydrogenase E2 component (PdhC; K00627), were selected for further analysis. Further, pyruvate decarboxylase (Pdc; K01568), acetyl-CoA synthetase (Acs; K01895), isocitrate/methylisocitrate lyase (Icl; K01637), and malate synthase (Mls; K01638) were selected as representative functions involved in the putative pathway from pyruvate to acetate and subsequently into the glyoxylate cycle. The enrichment analysis separately conducted on upregulated (log2FC > 1) and downregulated (log2FC < −1) DEGs identified K00101, K01895, K01637, and K01638 in the upregulated category, whereas K00162, K00627, and K01568 were determined in the downregulated category (Table S6). These results indicate activation of functions represented by Lldh, Acs, Icl, and Mls, whereas functions represented by PdhB, PdhC, and Pdc may be relatively suppressed under the Lac condition. In addition, genes encoding putative homologs of the *A. nidulans* lactate transporters JenA and JenB (AO090011000744 and AO090005000420), identified by BLASTP analysis [25], were also upregulated under the Lac condition (Table S4). The extracted KO terms and their expression changes were mapped to the corresponding metabolic pathways (Figure 3b). Lactate transport, conversion of lactate to pyruvate, and the glyoxylate cycle were likely activated under the Lac condition (Figure 3b, red), whereas carbon flux from pyruvate to the TCA cycle or the putative pathway from pyruvate to acetate was likely deactivated (Figure 3b, blue). Transcript levels do not necessarily reflect actual metabolic fluxes; thus, the present transcriptome data alone cannot identify the precise metabolic bottleneck. Therefore, the proposed limitation in carbon flux toward the TCA cycle remains speculative and should be regarded as a working hypothesis. However, the reduced expression of genes involved in pyruvate conversion, including PdhB, PdhC, and Pdc, indicates that downstream utilization of lactate-derived pyruvate may be limited under the Lac condition. Therefore, pyruvate metabolism and subsequent carbon supply to the TCA cycle may represent a potential constraint for efficient *A. oryzae* growth on lactate.

### 3.2. Enhancement of Lactate Assimilation by Supplementing Glucose and Other Sugars

Transcriptome analysis revealed that *A. oryzae* undergoes substantial metabolic reprogramming, particularly in the TCA cycle and glyoxylate cycle. This may lead to changes in the balance and insufficiency of carbon flux into the TCA cycle, potentially limiting the supply required for gluconeogenesis. These findings may represent metabolic constraints for lactate assimilation. Therefore, to alleviate these constraints, we assessed the effects of supplementing glucose. Supplementation of Lac medium with glucose (1 g/L) markedly altered the cultivation profile. Lactate consumption was promoted following glucose depletion during the early stages of cultivation in the Lac + 1 g/L Glu condition (Figure 2c). After 120 h, approximately 53.1% of the initial lactate was consumed, accompanied by the formation of mycelial pellets similar to those observed under the Glu condition, and the EDCW reached 1.8 ± 0.09 g/L. In comparison, the EDCW obtained with 5 g/L lactate alone and 1 g/L glucose alone were 0.4 ± 0.08 g/L and 0.7 ± 0.05 g/L, respectively. Therefore, the EDCW observed under the Lac + 1 g/L Glu condition exceeded the sum of that obtained with either carbon source alone, suggesting that at least a portion of the consumed lactate contributed to biomass production. These results reveal that glucose not only functions as an additional carbon source but also promotes physiological conditions that support lactate assimilation. A similar effect was observed when D-lactate was used instead of L-lactate (Figure S3). Furthermore, lactate assimilation was induced even at 0.2 g/L glucose (Figure S4A), indicating that only a small amount of glucose is sufficient to permit lactate assimilation in *A. oryzae*. Moreover, the extent of lactate consumption increased in a glucose concentration-dependent manner (Figure S4B). The EDCW observed under the Lac + 0.2 g/L Glu and Lac + 5 g/L Glu conditions exceeded the sum of that obtained with either carbon source alone (Figure S4C), supporting the contribution of assimilated lactate to biomass production.

To investigate the specificity of glucose in promoting lactate assimilation, various sugars were assessed as co-existing carbon sources. Promotion of lactate consumption was observed in the presence of α-L-rhamnose, glycerol, sucrose, D-fructose, D-xylose, starch, D-sorbitol, and maltose, with more than 40% of the initial lactate consumed within 72 h under each condition (Figure S5A). Further, fungal growth was improved in these cultures (Figure S5B). These findings indicate that the induction of lactate assimilation is not specific to glucose but can be broadly triggered by the presence of other carbon sources.

**Figure 4:**
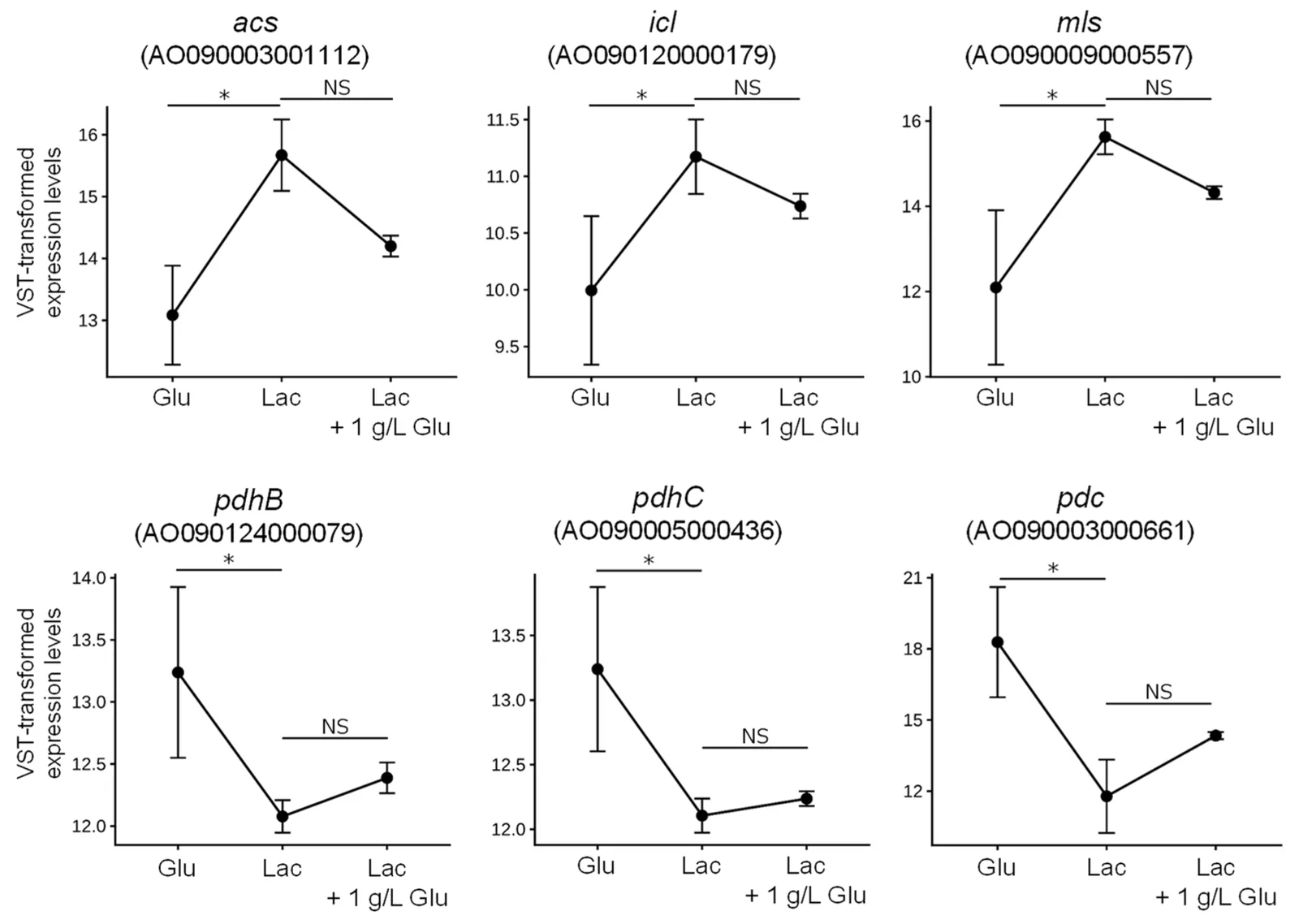
VST-transformed expression levels of genes corresponding to enzymatic functions shown in Figure 3b. Gene expression levels are shown as VST-transformed values. Statistical comparisons between Glu and Lac conditions, and between Lac and Lac + 1 g/L Glu conditions, were performed using DESeq2. Significance was identified based on adjusted *p*-values (*p*adj) obtained from differential expression analysis. Asterisks (*) indicate significant differences (*p*adj < 0.05), whereas NS indicates no significant difference.

To analyze the transcriptional changes associated with lactate assimilation, RNA-seq analysis was conducted using mycelia collected after 3 days of cultivation in Lac + 1 g/L Glu medium (Lac + 1 g/L Glu), a condition in which efficient lactate assimilation was observed in Figure 2c. The VST-transformed expression levels of genes associated with metabolic pathways potentially involved in lactate assimilation, as illustrated in Figure 3b, were compared among the Glu, Lac, and Lac + 1 g/L Glu conditions (Figure 4). When compared between the Glu and Lac conditions, genes annotated as Acs (*acs*, AO090003001112), Icl (*icl,* AO090120000179), and Mls (*mls*, AO090009000557) were significantly upregulated, whereas genes annotated as PdhB (*pdhB*, AO090124000079), PdhC (*pdhC*, AO090005000436), and Pdc (*pdc*, AO090003000661) were markedly downregulated under the Lac condition. These results were consistent with pathway enrichment analysis illustrated in Figure 3b. Under the Lac + 1 g/L Glu condition, *acs*, *icl*, and *mls* showed a decreasing trend that was not statistically significant. Conversely, *pdhB*, *pdhC*, and *pdc* increased in expression relative to the Lac condition, although these changes were not statistically significant. Overall, the expression profiles of genes involved in pyruvate metabolism and the glyoxylate cycle shifted toward those observed under the Glu condition. Interestingly, although glucose had been depleted by 72 h of cultivation and lactate had become the primary carbon source under the Lac + 1 g/L Glu condition, the expression profiles of these genes did not fully converge with those observed under the Lac condition.

### 3.3. Lactate Assimilation Is Enabled by Amino Acids

Transcriptome analysis and sugar supplementation experiments imply that additional physiological or metabolic factors are required to establish lactate-assimilating conditions in *A. oryzae*. We investigated the effects of amino acid addition on lactate assimilation to identify the medium components that may contribute to such conditions. Each of the 20 proteinogenic amino acids was individually added at a final concentration of 0.5 g/L, and their effects on lactate assimilation were assessed. Among the tested amino acids, arginine, proline, alanine, tyrosine, and glutamic acid markedly promoted lactate consumption during 72 h of cultivation, leading to the consumption of more than 40% of the initial lactate (Figure S6A). Fungal growth was also enhanced under these five conditions, with the EDCW increasing by approximately 1.2–1.8 g/L (Figure S6B). These results indicate that specific amino acids promote lactate assimilation in *A. oryzae*.

**Figure 5:**
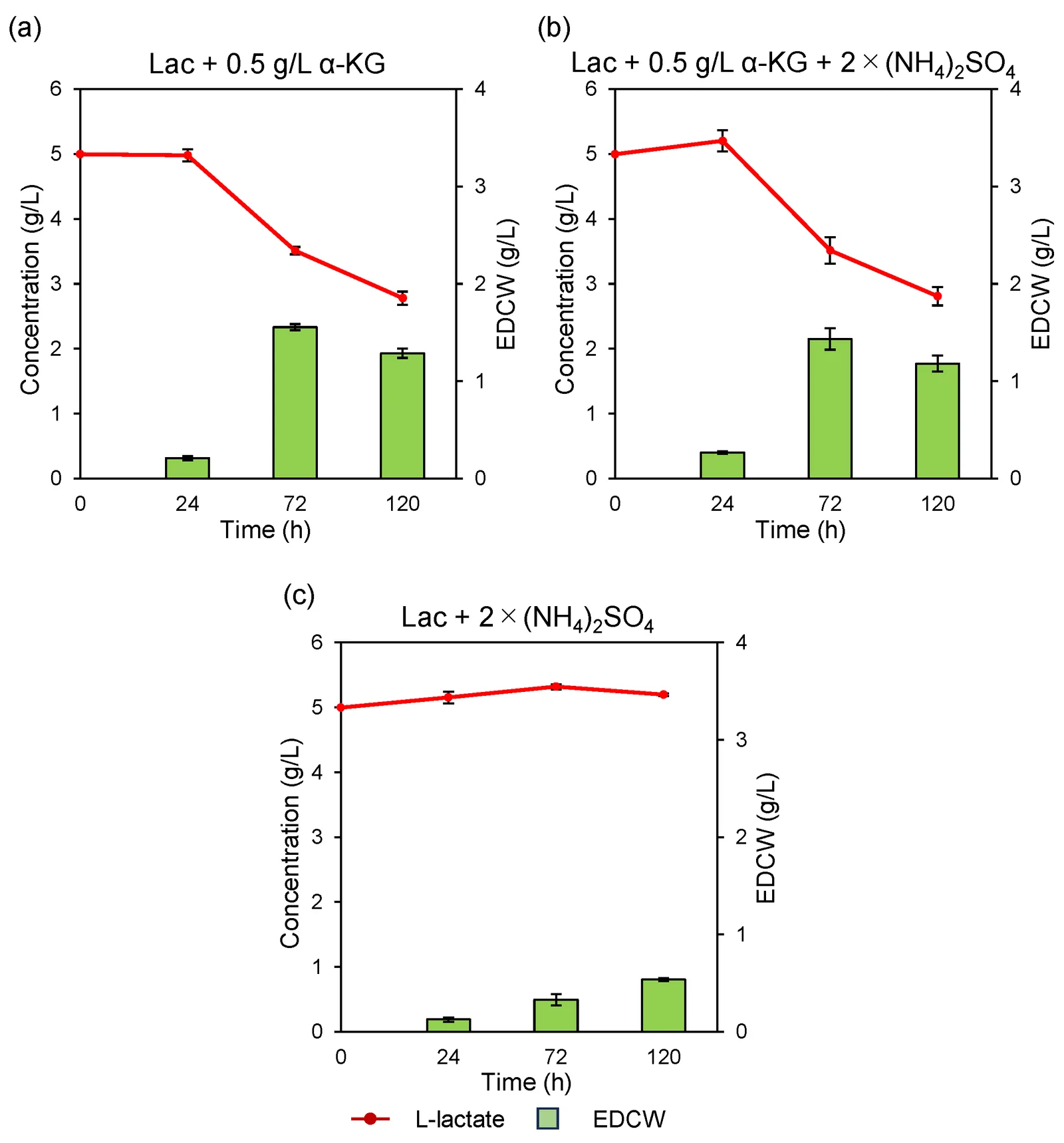
Effect of α-KG supplementation on lactate assimilation by *A. oryzae*. Time-course profiles of cell growth and lactate consumption under the following conditions: Lac + 0.5 g/L α-KG (**a**), Lac + 0.5 g/L α-KG + 2 × (NH_4_)_2_SO_4_ (**b**), and Lac + 2 × (NH_4_)_2_SO_4_ (**c**). Data are presented as mean ± SD of three biological replicates (*n* = 3). All other details are as described in Figure 2.

Arginine, proline, and glutamic acid are known to contribute to the supply of α-KG and ammonia (NH_3_) through amino acid metabolism [35]. *A. oryzae* was cultivated using Lac medium supplemented with 0.5 g/L α-KG (Lac + 0.5 g/L α-KG), Lac medium containing a twofold higher concentration of ammonium sulfate (Lac + 2 × (NH_4_)_2_SO_4_), and Lac medium containing both supplements (Lac + 0.5 g/L α-KG + 2 × (NH_4_)_2_SO_4_) to investigate the involvement of these metabolic contributions in lactate assimilation (Figure 5). Under Lac + 0.5 g/L α-KG and Lac + 0.5 g/L α-KG + 2 × (NH_4_)_2_SO_4_ conditions, a marked decrease in lactate concentration was observed, with 44.4% and 43.8% of the initial lactate consumed within 120 h, respectively (Figure 5a,b). Further, fungal growth was enhanced, reaching approximately 1.6 ± 0.03 g/L and 1.4 ± 0.1 g/L, respectively. Notably, comparable levels of lactate consumption and fungal growth were observed in the presence and absence of additional nitrogen, indicating that the effect of α-KG supplementation was largely independent of nitrogen availability. Indeed, increased ammonium sulfate concentrations alone had limited effects on lactate assimilation and fungal growth (Figure 5c).

### 3.4. Enhancement of Lactate Assimilation Under Low-pH Conditions

**Figure 6:**
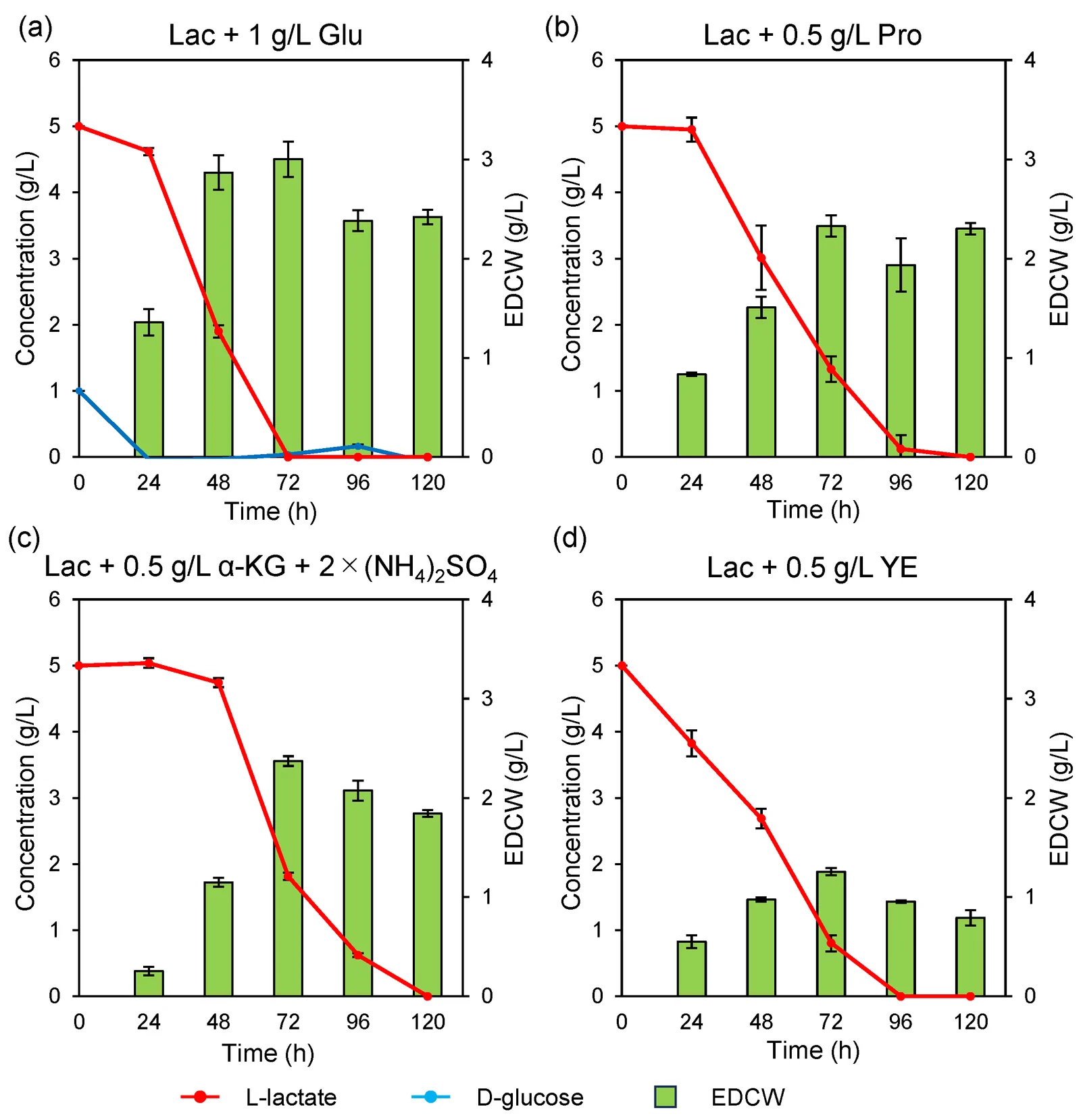
Effect of low-pH conditions on lactate assimilation by *A. oryzae*. Time-course profiles of fungal cell growth and carbon source consumption under the following conditions at an initial pH of 3.8: Lac + 1 g/L Glu, (**a**); Lac + 0.5 g/L Pro, (**b**); Lac + 0.5 g/L α-KG + 2 × (NH_4_)_2_SO_4_, (**c**); and Lac + 0.5 g/L YE, (**d**). Data are presented as mean ± SD of three biological replicates (*n* = 3). All other details are as described in Figure 2.

In previous experiments, the culture medium pH was adjusted to near-neutral conditions, which are commonly used for *A. oryzae* cultivation. To assess the effect of pH on lactate assimilation, cultures were prepared at an initial pH of 3.8 using Lac + 1 g/L Glu, Lac medium supplemented with 0.5 g/L proline (Lac + 0.5 g/L Pro), and Lac + 0.5 g/L α-KG + 2 × (NH_4_)_2_SO_4_ media. Under low-pH conditions, lactate assimilation and fungal growth were further improved (Figure 6a–c). In the Lac + 1 g/L Glu condition, lactate was completely consumed within 72 h, whereas only 53.1% of the initial lactate was consumed after 120 h under neutral pH conditions (Figure 2c). Mycelial pellets similar to those observed under neutral pH conditions were also formed, and EDCW reached 3.0 ± 0.2 g/L (Figure 6a), which was 1.7-fold higher than that obtained under neutral pH conditions at 72 h (Tukey’s HSD test, *p* = 0.0004). In the Lac + 0.5 g/L Pro (Figure 6b) and Lac + 0.5 g/L α-KG + 2 × (NH_4_)_2_SO_4_ conditions (Figure 6c), lactate was completely consumed within 120 h, with EDCW reaching 2.3 ± 0.1 g/L and 2.4 ± 0.05 g/L, respectively. Compared with the corresponding results obtained under neutral pH conditions (Figure 5b and Figure S6), both lactate consumption and fungal growth were substantially improved. These results indicate that low-pH conditions strongly facilitate lactate assimilation in *A. oryzae* under diverse nutritional conditions. Further, to explore a cost-effective strategy for industrial applications, YE was assessed as an alternative amino acid source. In the Lac medium supplemented with 0.5 g/L YE at pH 3.8 (Lac + 0.5 g/L YE), lactate was completely consumed within 120 h, and EDCW reached 1.3 ± 0.04 g/L (Figure 6d). This result indicates that YE is a practical amino acid source for supporting lactate assimilation by *A. oryzae*.

To evaluate whether the promotion of lactate assimilation by co-substrate addition and low pH was specific to the RIB40 strain, other *A. oryzae* strains (RIB128, RIB143, RIB163, RIB301, RIB306, RIB315, RIB915, RIB1108, RIB1172, RIB1178, and OIS1) were cultivated under the Lac at pH 5.8 and Lac + 1 g/L Glu at pH 3.8 conditions. As a result, lactate consumption was markedly promoted, and EDCW was substantially increased under the Lac + 1 g/L Glu (pH 3.8) condition compared with the Lac (pH 5.8) condition in all strains tested (Figure S7).

## 4. Discussion

In this study, we investigated culture conditions according to metabolic bottlenecks inferred by transcriptome analysis, which revealed that lactate assimilation in *A. oryzae* is promoted by supplementation with small amounts of sugars or specific amino acids (Figure 2c, Figures S5 and S6). Further, low-pH conditions improved lactate assimilation (Figure 6). These results indicate that lactate assimilation in *A. oryzae* is dependent on the metabolic state and environmental conditions.

Transcriptome analysis revealed that although lactate assimilation was not observed under the Lac condition, the expression of lactate transporters (JenA and JenB) and Lldh was upregulated (Figure 3b). This indicates that *A. oryzae* undergoes metabolic reprogramming in response to the presence of lactate, and that lactate uptake and its conversion to pyruvate are unlikely to represent a bottleneck for lactate assimilation at the transcriptional level (Figure 3b). Acs and glyoxylate cycle-related enzymes, Icl and Mls, were upregulated (Figure 3b). Conversely, PdhB and PdhC, which catalyze the major route from pyruvate to acetyl-CoA for entry into the TCA cycle, were downregulated. Further, Pdc was also downregulated, indicating that carbon flux from pyruvate toward acetate-derived acetyl-CoA and the glyoxylate cycle may likewise be reduced (Figure 3b). These results indicate that a metabolic state may be established in which carbon flux from pyruvate into the TCA cycle is limited [22,23]. Transcriptome analysis alone cannot determine enzyme activities or metabolic fluxes; therefore, the proposed mechanism remains speculative and should be interpreted with caution. Nevertheless, the reduced expression of Pdh- and Pdc-associated pathways implies that carbon flux from pyruvate toward the TCA cycle may be insufficient to support efficient growth. Consequently, maintenance of carbon flux into the TCA cycle, rather than lactate uptake itself, may represent a key requirement for efficient lactate assimilation. Future studies employing 13C-metabolic flux analysis will be required to validate this hypothesis.

The expression of PdhB, PdhC, and Pdc tended to increase relative to the Lac condition, whereas Acs, Icl, and Mls tended to decrease, in the Lac + 1 g/L Glu condition, where active lactate assimilation was observed (Figure 4). Notably, although these transcriptional changes were not statistically significant, the overall transcriptional profiles remained distinct from those observed under the Lac condition, despite glucose having already been depleted and lactate having become the primary carbon source. This observation may suggest that transient exposure to a small amount of glucose influences a metabolic state that persists during subsequent lactate assimilation. The increased expression of Pdh- and Pdc-associated genes together with the reduced expression of Acs-, Icl-, and Mls-associated pathways may be consistent with a shift toward sustained carbon flux into the TCA cycle and reduced reliance on alternative carbon-conserving pathways. Altogether, these observations may suggest that efficient lactate assimilation is associated with a metabolic state that maintains carbon flux into the TCA cycle. 

In *S. cerevisiae*, the expression of genes involved in the utilization of non-fermentable carbon sources, including gluconeogenesis, the glyoxylate cycle, and the lactate transporter gene *jen1*, is regulated by transcriptional regulatory mechanisms involving the Snf1 kinase, the transcriptional repressors Mig1/Mig2, and the transcriptional activator Cat8 [22,23]. Interestingly, putative homologs of *mig1*/*mig2* (K11215, AO090009000638) and *cat8* (no KO assigned, AO090012000525), predicted based on BLASTP analysis, were also identified as DEGs and were upregulated under the Lac condition (Table S4). These observations suggest that transcriptional responses observed during lactate utilization may be associated with regulatory mechanisms similar to those reported in yeast. However, the involvement of these regulatory factors remains hypothetical, and further studies, such as analyses using transcription factor-disruption strains, will be required to validate their roles.

The observation that α-KG supplementation promoted lactate assimilation is consistent with, although it does not directly demonstrate, the hypothesis that the availability of TCA-cycle intermediates influences lactate assimilation. Because α-KG directly enters the TCA cycle, its supplementation may alleviate limitations in carbon supply to central metabolism and thereby facilitate the use of lactate-derived pyruvate (Figure 5) [35]. Notably, amino acids that promote lactate assimilation, including glutamate, proline, and arginine, are metabolically associated with α-ketoglutarate production. Together with the positive effect of direct α-ketoglutarate supplementation, these observations indicate that lactate assimilation enhancement does not depend on a specific compound itself, but rather on metabolic processes that improve carbon supply to the TCA cycle.

Furthermore, this study revealed that low-pH conditions (pH 3.8) were favorable for lactate assimilation (Figure 6), although *A. oryzae* is typically cultivated under near-neutral conditions (approximately pH 6.5). The effect of pH was consistently observed across all supplement conditions, indicating that low pH may act as an independent environmental factor. The pKa of lactate is 3.86 [36], and the proportion of the undissociated form increases under low-pH conditions, which may improve membrane permeability and facilitate cellular uptake [37]. Therefore, enhanced lactate uptake is one possible explanation for the observed improvement in lactate assimilation. However, the present study did not measure intracellular lactate concentrations or evaluate transporter activity, and thus this possibility remains to be experimentally verified. Future studies will be required to validate this hypothesis. In addition, low pH may influence not only lactate transport but also broader aspects of fungal physiology and gene expression [38]. Therefore, these potential effects should also be considered when interpreting the promotion of lactate assimilation under low-pH conditions. Consequently, the mechanism underlying the promotion of lactate assimilation under low-pH conditions remains unclear, although the present results clearly demonstrate that low pH enhances lactate assimilation in *A. oryzae*.

Overall, the transcriptome and cultivation data suggest that efficient lactate assimilation may be associated with a metabolic state favorable for maintaining central carbon metabolism, including carbon flux into the TCA cycle. Environmental factors, such as low pH, were also found to further improve this process, although the underlying mechanism remains unclear. The promotion of lactate assimilation under Lac + 1 g/L Glu and pH 3.8 conditions was observed in multiple *A. oryzae* strains, indicating a common physiological trait (Figure S7). However, the proposed bottlenecks for lactate assimilation predicted through transcriptome analysis and culture experiments remain hypothetical. In the future, based on the findings of this study, 13C-metabolic flux analysis and gene disruption approaches are expected to reveal the metabolic mechanisms of lactate assimilation in *A. oryzae*, leading to further improvements.

## 5. Conclusions

The present results expand our understanding of lactate utilization in *A. oryzae* and provide a basis for developing bioprocesses that use lactate-rich byproducts and fermentation residues. Such resources are generated in various food and fermentation industries, including dairy processing, probiotic production, and lactic acid fermentation processes, and are attracting increasing attention as substrates for microbial upcycling. This study broadens the range of feedstocks that can potentially be used by *A. oryzae* by identifying culture conditions that enable efficient lactate assimilation. *A. oryzae* is considered a safe microorganism and is increasingly being explored as a host for edible biomass production, enzyme production, and other value-added bioprocesses; thus, improved lactate assimilation may facilitate the conversion of lactate-rich resources into fungal biomass and other valuable products. Furthermore, efficient utilization of residual lactate could contribute to reducing the organic load of fermentation residues while simultaneously generating useful biomass, thereby supporting the development of sustainable bioprocesses for food byproduct valorization and circular bioeconomy applications.

## Data Availability

The datasets generated and/or analyzed during the current study are available from the corresponding author upon reasonable request. RNA-seq data supporting the results of this study were deposited in the DRA under the BioProject accession number PRJDB42398.

## Supplementary Materials

The following supporting information can be downloaded at https://www.mdpi.com/article/10.3390/jof12090679/s1: Figure S1: Calibration curve for the estimated dry cell weight (EDCW); Figure S2: KEGG pathway enrichment analysis using all DEGs between the Glu and Lac conditions; Figure S3: Effect of glucose supplementation on D-lactate assimilation by *A. oryzae*; Figure S4: Effect of glucose concentration on lactate assimilation by *A. oryzae*; Figure S5: Effect of various sugars on lactate assimilation by *A. oryzae*; Figure S6: Effect of various amino acids on lactate assimilation by *A. oryzae*; Figure S7: Effects of glucose supplementation and low pH on lactate assimilation in multiple *A. oryzae* strains; Table S1: Gene count matrix for RNA-seq under different carbon source conditions; Table S2: Correspondence between *A. oryzae* genes and KEGG orthologs; Table S3: Results of differentially expressed analysis between Glu and Lac conditions; Table S4: DEGs between Glu and Lac conditions; Table S5: KEGG pathway enrichment analysis using all DEGs between the Glu and Lac conditions; Table S6: KEGG orthologs, direction classification, and functional annotations in three selected enriched pathways.

## Abbreviations

The following abbreviations are used in this manuscript:

α-KG	α-ketoglutarate
A_260_	Absorbance at 260 nm
CD	Czapek–Dox
DEGs	Differentially expressed genes
EDCW	Estimated dry cell weight
EDTA·2Na	Disodium ethylenediaminetetraacetate
FDR	False discovery rate
FeSO_4_·7H_2_O	Ferrous sulfate heptahydrate
H_3_BO_3_	Boric acid
HPLC	High-performance liquid chromatography
KEGG	Kyoto Encyclopedia of Genes and Genomes
KO	KEGG ortholog
log_2_FC	log_2_ fold change
MgSO_4_·7H_2_O	Magnesium sulfate heptahydrate
MnCl_2_·4H_2_O	Manganese(II) chloride tetrahydrate
(NH_4_)_6_Mo_7_O_24_·4H_2_O	Ammonium heptamolybdate tetrahydrate
TCA	Tricarboxylic acid
VST	Variance-stabilizing transformation
YE	Yeast extract
ZnSO_4_·7H_2_O	Zinc sulfate heptahydrate
CoCl_2_·6H_2_O	Cobalt(II) chloride hexahydrate
CuSO_4_·5H_2_O	Copper(II) sulfate pentahydrate

## References

[1] Ortiz-Sanchez M., Inocencio-García P.-J., Alzate-Ramírez A.F., Cardona Alzate C.A. (2023). Potential and restrictions of food-waste valorization through fermentation processes. Fermentation.

[2] Gonçalves E.M., Pestana J.M., Alvarenga N. (2026). Fermenting the unused: Microbial biotransformation of food industry by-products for circular bioeconomy valorisation. Fermentation.

[3] Taylor M.J., Richardson T. (1979). Applications of microbial enzymes in food systems and in biotechnology. Adv. Appl. Microbiol..

[4] U.S. Food and Drug Administration Microorganisms & Microbial-Derived Ingredients Used in Food (Partial List). https://www.fda.gov/food/generally-recognized-safe-gras/microorganisms-microbial-derived-ingredients-used-food-partial-list.

[5] Naeem M., Manzoor S., Abid M.-U.-H., Tareen M.B.K., Asad M., Mushtaq S., Ehsan N., Amna D., Xu B., Hazafa A. (2022). Fungal proteases as emerging biocatalysts to meet the current challenges and recent developments in biomedical therapies: An updated review. J. Fungi.

[6] Tanaka M., Gomi K. (2021). Induction and repression of hydrolase genes in *Aspergillus oryzae*. Front. Microbiol..

[7] Machida M., Asai K., Sano M., Tanaka T., Kumagai T., Terai G., Kusumoto K., Arima T., Akita O., Kashiwagi Y. (2005). Genome sequencing and analysis of *Aspergillus oryzae*. Nature.

[8] Jin F.-J., Hu S., Wang B.-T., Jin L. (2021). Advances in genetic engineering technology and its application in the industrial fungus *Aspergillus oryzae*. Front. Microbiol..

[9] Sun Z., Wu Y., Long S., Feng S., Jia X., Hu Y., Ma M., Liu J., Zeng B. (2024). *Aspergillus oryzae* as a cell factory: Research and applications in industrial production. J. Fungi.

[10] Terramino, Inc. dba Prime Roots. https://www.primeroots.com/.

[11] Formo Bio GmbH. https://formo.bio/process/.

[12] Souza Filho P.F., Nair R.B., Andersson D., Lennartsson P.R., Taherzadeh M.J. (2018). Vegan-mycoprotein concentrate from pea-processing industry byproduct using edible filamentous fungi. Fungal Biol. Biotechnol..

[13] Nakamura M., Hattori R., Suzuki S., Mano J. (2025). Upcycling whey through the production of edible *Aspergillus oryzae* cell biomass. Biosci. Biotechnol. Biochem..

[14] Devanthi P.V.P., Pratama F., Pramanda I.T., Bani M.D., Kadar A.D., Kho K. (2024). Exploring the potential of *Aspergillus oryzae* for sustainable mycoprotein production using Okara and Soy Whey as cost-effective substrates. J. Fungi.

[15] Rocha-Mendoza D., Kosmerl E., Krentz A., Zhang L., Badiger S., Miyagusuku-Cruzado G., Mayta-Apaza A., Giusti M., Jiménez-Flores R., García-Cano I. (2021). Invited review: Acid whey trends and health benefits. J. Dairy Sci..

[16] Loo J.S., Oslan S.N.H., Mokshin N.A.S., Othman R., Amin Z., Dejtisakdi W., Prihanto A.A., Tan J.S. (2025). Comprehensive review of strategies for lactic acid bacteria production and metabolite enhancement in probiotic cultures: Multifunctional applications in functional foods. Fermentation.

[17] Canibe N., Jensen B.B. (2012). Fermented liquid feed—Microbial and nutritional aspects and impact on enteric diseases in pigs. Anim. Feed Sci. Technol..

[18] Lo S.C., Yang C.Y., Mathew D.C., Huang C.C. (2021). Growth and autolysis of the kefir yeast *Kluyveromyces marxianus* in lactate culture. Sci. Rep..

[19] Mansour S., Beckerich J.M., Bonnarme P. (2008). Lactate and amino acid catabolism in the cheese-ripening yeast *Yarrowia lipolytica*. Appl. Environ. Microbiol..

[20] Pacheco A., Talaia G., Sá-Pessoa J., Bessa D., Gonçalves M.J., Moreira R., Paiva S., Casal M., Queirós O. (2012). Lactic acid production in *Saccharomyces cerevisiae* is modulated by expression of the monocarboxylate transporters Jen1 and Ady2. FEMS Yeast Res..

[21] Garvie E.I. (1980). Bacterial lactate dehydrogenases. Microbiol. Rev..

[22] Schüller H.-J. (2003). Transcriptional control of nonfermentative metabolism in the yeast *Saccharomyces cerevisiae*. Curr. Genet..

[23] Turcotte B., Liang X.B., Robert F., Soontorngun N. (2010). Transcriptional regulation of nonfermentable carbon utilization in budding yeast. FEMS Yeast Res..

[24] Li Y., Cao Y., He B., Liu X., Ai M., Tu Y. Molecular cloning, expression and characterization of a novel L-lactate dehydrogenase from *Aspergillus oryzae*. Proceedings of the 2020 9th International Conference on Bioinformatics and Biomedical Science (ICBBS 2020).

[25] Sá-Pessoa J., Amillis S., Casal M., Diallinas G. (2015). Expression and specificity profile of the major acetate transporter AcpA in *Aspergillus nidulans*. Fungal Genet. Biol..

[26] Torres A., Li S.M., Roussos S., Vert M. (1996). Screening of microorganisms for biodegradation of poly(Lactic-Acid) and lactic acid-containing polymers. Appl. Environ. Microbiol..

[27] Mahboubi A., Ferreira J., Taherzadeh M., Lennartsson P. (2017). Production of fungal biomass for feed, fatty acids, and glycerol by *Aspergillus oryzae* from fat-rich dairy substrates. Fermentation.

[28] Kikuta H., Sushida H., Tanaka T., Kotake E., Tsuzuki W., Hattori R., Suzuki S., Kusumoto K.-I., Mano J. (2025). α-linolenic acid production in *Aspergillus oryzae* via the overexpression of an endogenous omega-3 desaturase gene. Fermentation.

[29] Ouchi K., Ishido T., Sugama S., Nojiro K. (1967). Studies on the ecology of yeast in koji. III. Measurement of koji mold growth during koji making. J. Brew. Soc. Jpn..

[30] Ewels P.A., Garcia M.U., Alneberg J., Nahnsen S., Wilm A., Di Tommaso P., Peltzer A., Fillinger S., Patel H. (2020). The Nf-core framework for community-curated bioinformatics pipelines. Nat. Biotechnol..

[31] Love M.I., Huber W., Anders S. (2014). Moderated estimation of fold change and dispersion for RNA-Seq data with DESeq2. Genome Biol..

[32] Wu T., Hu E., Xu S., Chen M., Guo P., Dai Z., Feng T., Zhou L., Tang W., Zhan L. (2021). ClusterProfiler 4.0: A universal enrichment tool for interpreting omics data. Innovation.

[33] Yu G. (2025). Enrichplot: Visualization of Functional Enrichment Result.

[34] Nakatsukasa K., Nishimura T., Byrne S.D., Okamoto M., Takahashi-Nakaguchi A., Chibana H., Okumura F., Kamura T. (2015). The ubiquitin ligase SCFUcc1 acts as a metabolic switch for the glyoxylate cycle. Mol. Cell.

[35] Owen O.E., Kalhan S.C., Hanson R.W. (2002). The key role of anaplerosis and cataplerosis for citric acid cycle function. J. Biol. Chem..

[36] George Washington University Table of Acids with Kas and pKas. https://library.gwu.edu/sites/default/files/2023-12/Table%20of%20Acids%20w%20Kas%20and%20pKas.pdf.

[37] Casal M., Paiva S., Queirós O., Soares-Silva I. (2008). Transport of carboxylic acids in yeasts. FEMS Microbiol. Rev..

[38] Peñalva M.A., Tilburn J., Bignell E., Arst H.N. (2008). Ambient pH gene regulation in fungi: Making connections. Trends Microbiol..

