# Supplementary material for: Enhancing Lactate Assimilation in Aspergillus oryzae by Small Amounts of Metabolic Co-Substrates and Low pH
Source: J Fungi (Basel). 2026 Sep 10;12(9):679. doi: 10.3390/jof12090679 (PMC13608414; doi:10.3390/jof12090679)
Supplement: Supplementary file 1 [file jof-12-00679-s001.zip › Lactic_acid_metabolism_sup_20260902.pdf]

**Supplementary figures**

Hiroki Kikuta [1,†], Masamichi Muto [2,†], Naoko Tanaka [2], Tsuyoshi Tanaka [3,4], and Junichi Mano [1,5*]

[1] Institute of Food Research, National Agriculture and Food Research Organization, 2-1-12 Kannondai, Tsukuba, Ibaraki 305-8642, Japan
[2] Microbial Technology Research Section, Biotics Research Institute, R&D Division, Morinaga Milk Industry Co., Ltd., 5-1-83 Higashihara, Zama, Kanagawa 252-8583, Japan
[3] Research Center for Advanced Analysis, National Agriculture and Food Research Organization, 2-1-2 Kannondai, Tsukuba, Ibaraki 305-8518, Japan
[4] Institute of Crop Science, National Agriculture and Food Research Organization, 2-1-2 Kannondai, Tsukuba, Ibaraki 305-8518, Japan
[5] Faculty of Life and Environmental Sciences, University of Tsukuba, 1-1-1 Tennodai, Tsukuba, Ibaraki 305–8572, Japan
* Correspondence: mano.junichi544@naro.go.jp; Tel.: +81-29-838-7321
[†] These authors contributed equally to this study

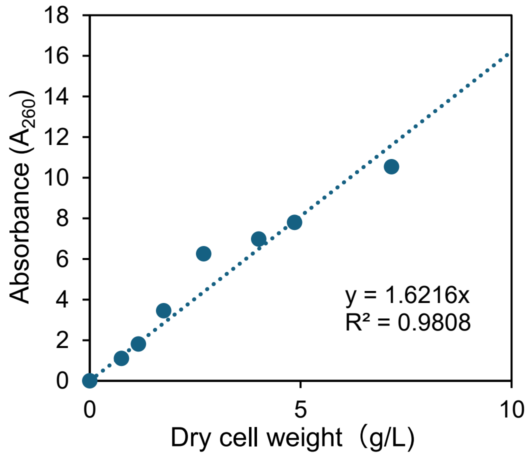


**Figure S1. Calibration curve for the estimated dry cell weight (EDCW)**

Freeze-dried *A. oryzae* biomass suspensions (0-10 g/L) were extracted for nucleic acid by perchloric acid extraction. The biomass was obtained from cultures grown in 2 mL of CD medium at 30 °C and 200 rpm for 2 days. The nucleic acid content was quantified by absorbance at 260 nm ($A_{260}$). The relationship between the dry cell weight and $A_{260}$ was used to estimate dry cell weight (EDCW) in subsequent experiments. A linear regression constrained to pass through the origin gave A260 = 1.6216 × dry cell weight (g/L) ($R^2$ = 0.9808).

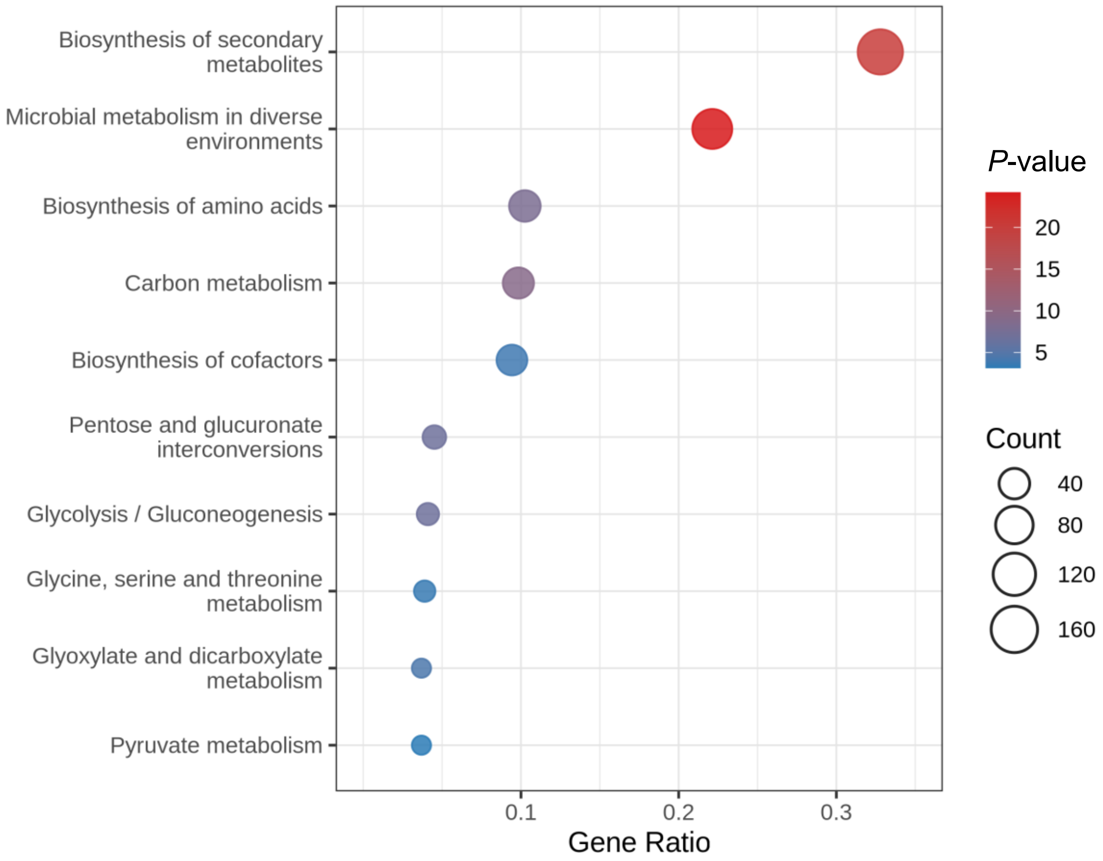


**Figure S2. KEGG pathway enrichment analysis using all DEGs between the Glu and Lac conditions.**

Significantly enriched KEGG pathways are shown. The X-axis indicates the GeneRatio, representing the proportion of KO terms classified into each KEGG pathway among the KO terms corresponding to the DEGs used for the enrichment analysis. For example, the pathway "Biosynthesis of secondary metabolites" contains 160 KO terms, accounting for approximately 33% of the 488 KO terms included in the analysis, resulting in a GeneRatio of 0.33. A higher GeneRatio indicates that a larger proportion of the DEGs between the Glu and Lac conditions were assigned to a given pathway. The Y-axis indicates the top 10 significantly enriched pathways ranked by GeneRatio. Dot size corresponds to the number of KO terms classified into each pathway (Count). Dot color indicates the false discovery rate (FDR)-adjusted *P*-value, with lower FDR values indicating stronger statistical support that the observed enrichment is unlikely to have occurred by chance.

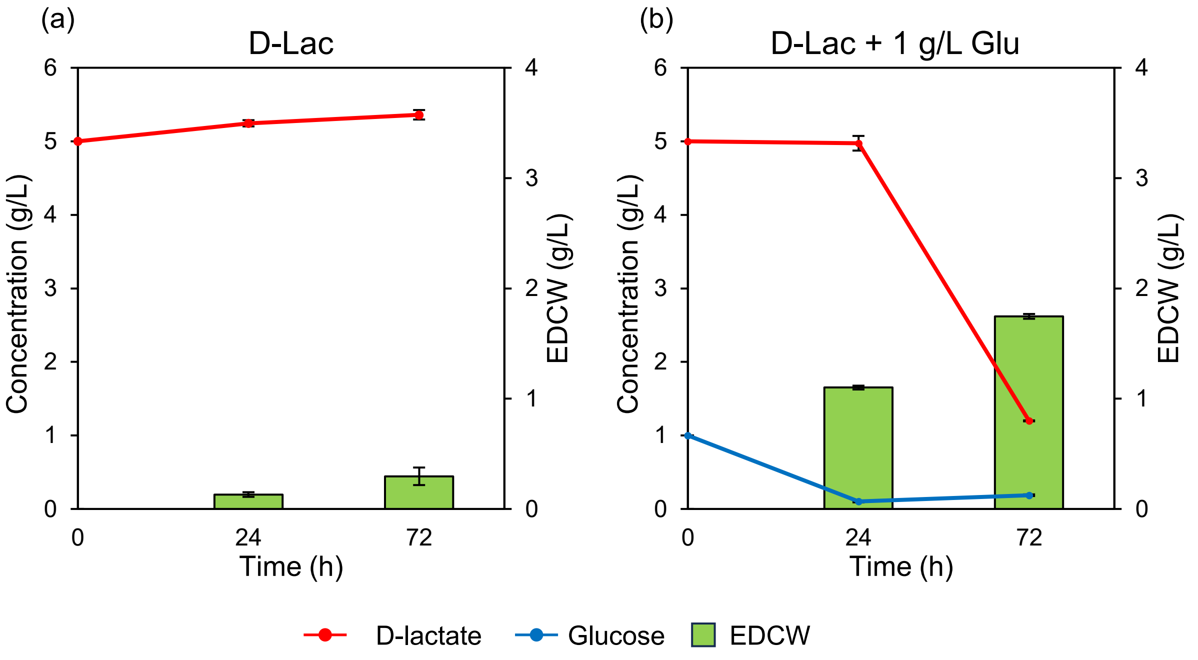


**Figure S3. Effect of glucose supplementation on D-lactate assimilation by *A. oryzae*.**

Time-course profiles of cell growth and carbon source consumption by *A. oryzae* under a medium containing 5 g/L D-lactate (D-Lac) as the sole carbon source (a) and the D-Lac medium supplemented with 1 g/L glucose (b). The green bars indicate EDCW, and lines indicate residual carbon source concentrations. Blue and red lines represent residual glucose and lactate concentrations, respectively. Data are presented as mean ± SD of three biological replicates (n = 3).

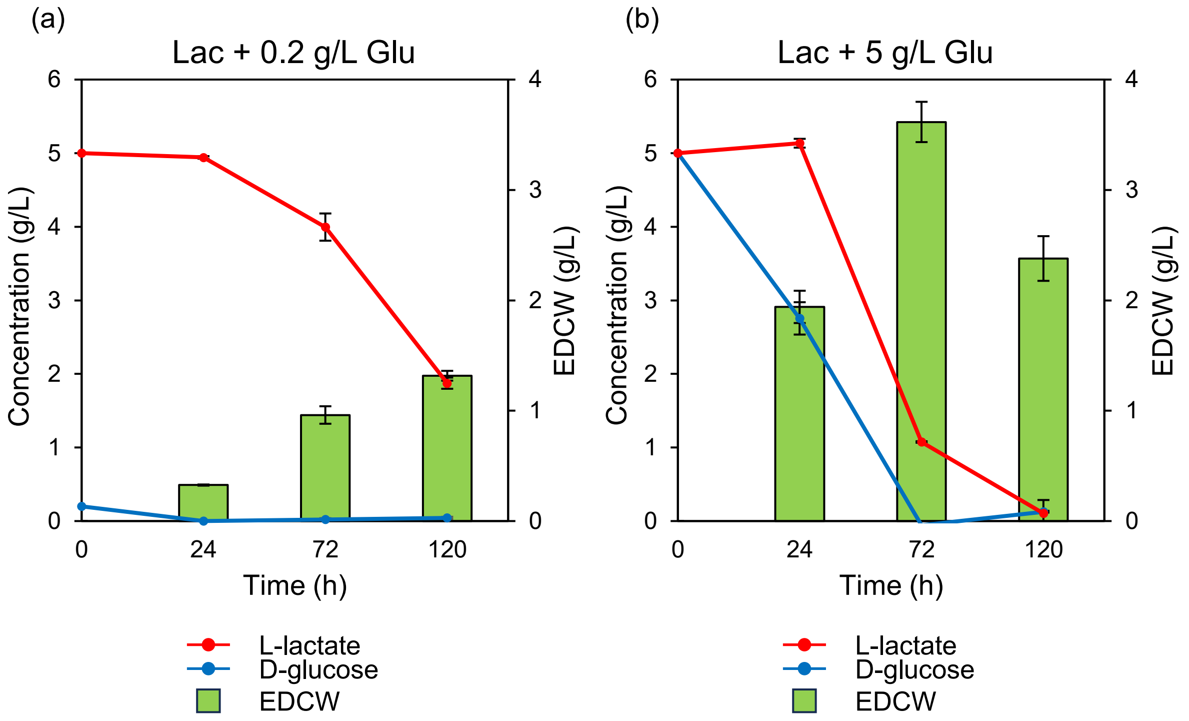


(c)

| Condition | Maximum EDCW (g/L) |
|---|---|
| Lac | 0.4 ± 0.08 |
| 0.2 g/L Glu | 0.3 ± 0.02 |
| Lac + 0.2 g/L Glu | 1.3 ± 0.04 |
| 5 g/L Glu | 2.4 ± 0.05 |
| Lac + 5 g/L Glu | 3.6 ± 0.2 |

**Figure S4. Effect of glucose concentration on lactate assimilation by *A. oryzae*.**

Time-course profiles of cell growth and carbon source consumption by *A. oryzae* under the Lac medium supplemented with 0.2 g/L (a) or 5 g/L (b) glucose. The green bars indicate EDCW, and lines indicate residual carbon source concentrations. Blue and red lines represent residual glucose and lactate concentrations, respectively. Comparison table of the maximum EDCW observed during the cultivation period under each condition (c). The 0.2 g/L Glu and 5 g/L Glu conditions contained glucose as the sole carbon source at the indicated concentrations. Maximum EDCW values represent the highest EDCW recorded during the cultivation period for each condition, which was observed at 48 h for the 0.2 g/L Glu condition and at 72 h for the 5 g/L Glu condition. Data are presented as mean ± SD of three biological replicates (n = 3).

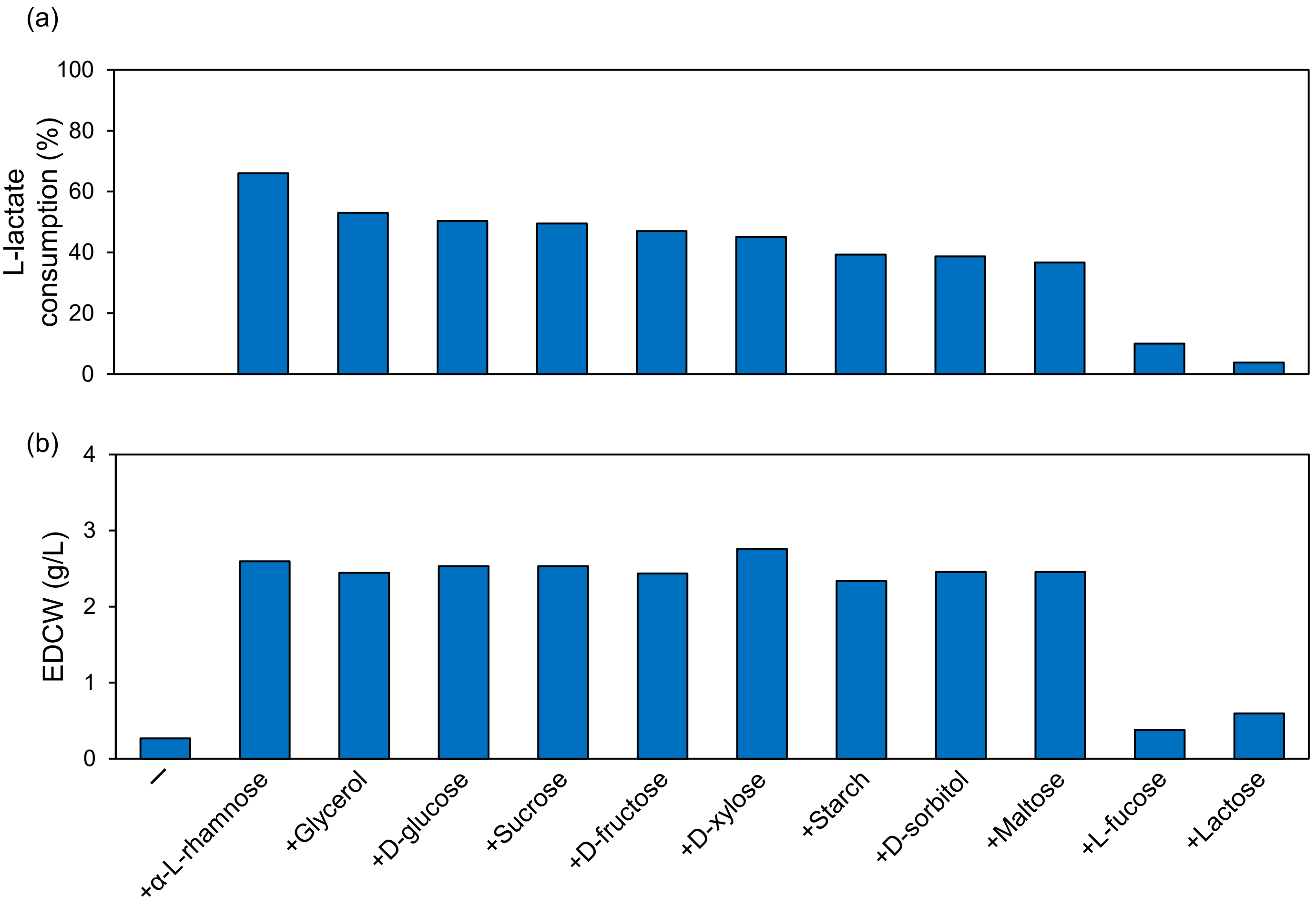


**Figure S5. Effect of various sugars on lactate assimilation by *A. oryzae*.**

*A. oryzae* was cultivated for 72 h in Lac medium supplemented with 1 g/L of glucose or other sugars, respectively. The lactate consumption ratio (%) (a) and EDCW (g/L) (b) are shown. Representative sugars that strongly promoted lactate assimilation and those with little or no effect are shown. The supplemented sugars are indicated below the figure. (−) represents the Lac medium without supplementation. Data represent a single biological replicate (n = 1).

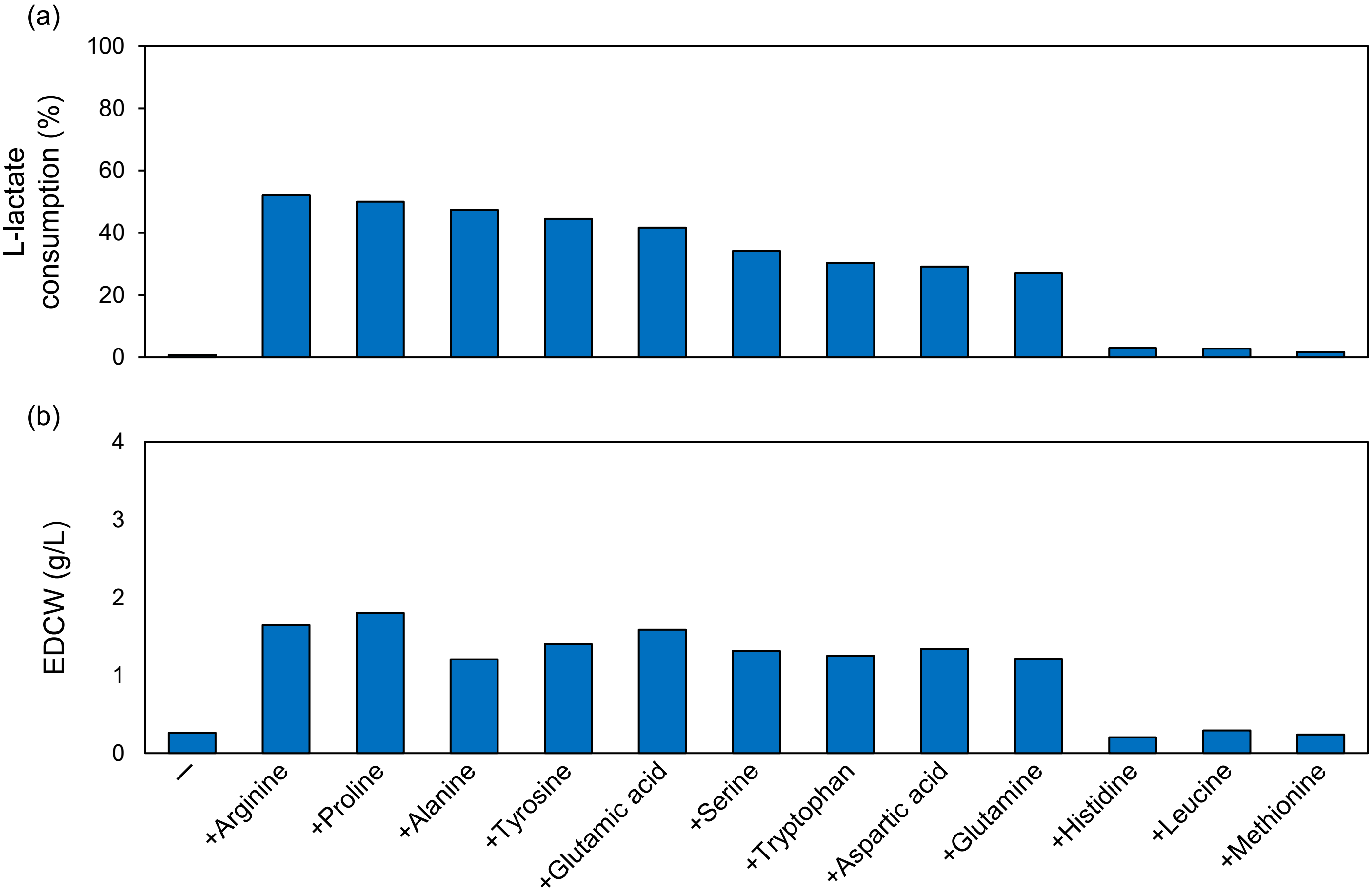


**Figure S6. Effect of various amino acids on lactate assimilation by *A. oryzae*.**

*A. oryzae* was cultivated for 72 h in Lac medium supplemented with 0.5 g/L of various amino acids, respectively. The lactate consumption ratio (%) (a) and EDCW (g/L) (b) are shown. Representative amino acids that strongly promoted lactate assimilation and those with little or no effect are shown. The supplemented amino acids are indicated below the figure. (−) represents the Lac medium without supplementation. Data represent a single biological replicate (n = 1).

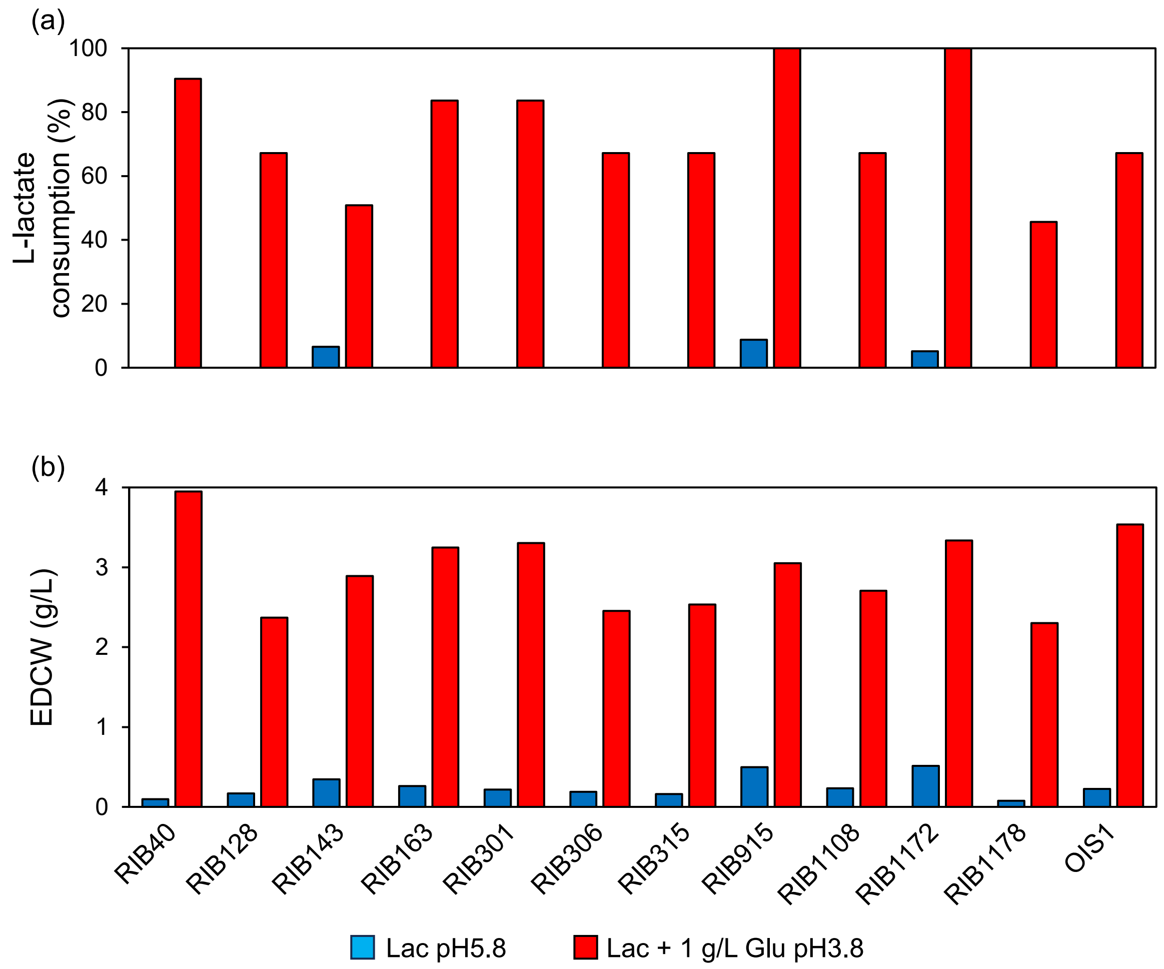


**Figure S7. Effects of glucose supplementation and low pH on lactate assimilation in multiple *A. oryzae* strains**.

Multiple *A. oryzae* strains were cultivated under either the Lac condition (initial pH 5.8, blue) or the Lac + 1 g/L Glu condition (initial pH 3.8, red). Lactate consumption ratio (%) (a) and EDCW (b) after 72 h of cultivation are shown. Data represent a single biological replicate (n = 1).
